# Renal Diffusion-Weighted Imaging in Healthy Dogs: Reproducibility, Test-Retest Repeatability, and Selection of the Optimal b-value Combination

**DOI:** 10.3389/fvets.2021.641971

**Published:** 2021-07-02

**Authors:** Sang-Kwon Lee, Juryeoung Lee, Seolyn Jang, Eunji Lee, Chang-Yeop Jeon, Kyung-Seoub Lim, Yeung Bae Jin, Jihye Choi

**Affiliations:** ^1^College of Veterinary Medicine and BK21 Plus Project Team, Chonnam National University, Gwangju, South Korea; ^2^National Primate Research Center, Korea Research Institute of Bioscience and Biotechnology, Cheongju, South Korea; ^3^Futuristic Animal Resource and Research Center, Korea Research Institute of Bioscience and Biotechnology, Cheongju, South Korea; ^4^College of Veterinary Medicine, Gyeongsang National University, Jinju, South Korea

**Keywords:** canine, diffusion-weighted magnetic resonance imaging, kidney, renal fibrosis, b-value

## Abstract

Diffusion-weighted imaging (DWI) magnetic resonance imaging can evaluate alterations in the microstructure of the kidney. The purpose of this study was to assess the apparent diffusion coefficient (ADC) and the intravoxel incoherent motion model (IVIM) parameters of a normal kidney in healthy dogs, to evaluate the effect of b-value combinations on the ADC value, and the reproducibility and test-retest repeatability in monoexponential and IVIM analysis. In this experimental study, the ADC, pure diffusion coefficient (D), pseudodiffusion coefficient (D^*^), and perfusion fraction (*f*_p_) were measured from both kidneys in nine healthy beagles using nine b-values (b = 0, 50, 70, 100, 150, 200, 500, 800, and 1,000 s/mm^2^) twice with a 1-week interval between measurements. Interobserver and intraobserver reproducibility, and test-retest repeatability of the measurements were calculated. ADC values were measured using 10 different b-value combinations consisting of three b-values each, and were compared to the ADC obtained from nine b-values. All the ADC, D, D^*^, and *f*_p_ values measured from the renal cortex, medulla, and the entire kidney had excellent interobserver and intraobserver reproducibility, and test-retest repeatability. The ADC obtained from a b-value combination of 0, 100, and 800 s/mm^2^ had the highest intraclass correlation coefficient with the ADC from nine b-values. The results of this study indicated that DWI MRI using multiple b-values is feasible for the measurement of ADC and IVIM parameters with high reproducibility and repeatability in the kidneys of healthy dogs. A combination of b = 0, 100, and 800 s/mm^2^ can be used for ADC measurements when multiple b-values are not available in dogs.

## Introduction

In veterinary medicine, the diagnosis and staging of chronic kidney disease (CKD) are based on the detection of structural change and renal dysfunction (1, 2). The International Renal Interest Society (IRIS) developed the guidelines for the staging of CKD based on the serum creatinine concentration, proteinuria, and systemic blood pressure. Recently, Symmetric dimethylarginine is routinely used as a more sensitive biomarker than creatinine in estimating GFR. However, these biochemical markers are insensitive to early renal injury and they can only assess the global renal function but not insight of the morphological change of the kidney.

Renal fibrosis is the histologic hallmark of CKD. It is final pathway in the progression of chronic kidney disease regardless of the initial insult and lead to irreversible kidney damage occurs and the kidney decrease in size (3). Therefore, the evaluation of the presence and degree of renal fibrosis may early diagnosis CKD, assess the severity of CKD, and provide prognostic information (4–6). A biopsy is required to evaluate renal fibrosis however this is difficult to perform in patients due to its high invasiveness and complications. Moreover, renal biopsy is not a suitable technique to monitor the progression of the disease and is prone to sampling errors. Although the severity of renal fibrosis in cats with CKD was higher in the later stage than in the early stage, each IRIS stage was no consistent with severity of renal fibrosis (7).

Diffusion-weighted imaging (DWI) magnetic resonance imaging (MRI) can estimate and quantify the diffusion of water molecule within the tissue. Accumulation of collagen and other matrix components in renal fibrosis restricts the diffusion of water molecules (3, 8). DWI assesses restricted diffusion and allows to estimate the renal fibrosis. On previous humans and animal studies, DWI reflected histological change in the renal interstitium such as renal fibrosis and cell density in various diseases including CKD, renal artery stenosis, and unilateral ureteral obstruction, thus recently DWI emerges as a potential imaging biomarker for renal fibrosis (5, 6, 9–11). Besides, DWI reflected split glomerular filtration rate in human patients with chronic nephropathy and renal stenosis artery (12). Therefore, DWI may determine the progression of CKD in each kidney and predict renal dysfunction in CKD patients, and several studies suggested the clinical feasibility of DWI for diagnosis and staging of CKD in humans (4, 6, 9, 13).

DWI quantify the magnitude of diffusion of water molecules within a tissue based on the signal decay according to the diffusion-sensing gradient pulse and provide clinically available measurements such as the apparent diffusion coefficient (ADC). When performing DWI, the operator should select a diffusion-weighting factor called “b-value” which involves the amplitude and duration of diffusion-sensing gradient pulse and the time between the gradient pulse (14). The selection of b-value determines the sensitivity of the DWI sequence to water diffusion. A high b-value in DWI provides better contrast by improving the sensitivity to tissue diffusivity; however, for high b-values, the signal-to-noise ratio is decreased as the larger diffusion gradient increases signal decays compared to those with low b-values (14). In contrast, if only a low b-value is used for a high signal-to-noise ratio, the contrast for diffusion may decrease. The selection of the b-value also contributes to the property of the signal decay curve and subsequently for diffusion measurement. The signal decay is not only affected by tissue diffusion but also by microcirculation during DWI (15–17). Because the signal decay by microcirculation is 10 times faster than tissue diffusion, this effect is mainly observed at low b-values (4). Therefore, in low b-values, the signal decay curve is steeper than the actual diffusion signal decay (15–17). In contrast, when the b-value increases, the rate of diffusion-related signal decay decreases due to non-Gaussian diffusion which is caused by the interaction of water molecules with obstacles such as cell membranes. Therefore, for high b-values, the signal decay curve becomes flattened compared to that for low b-values. Additionally, the background may mimic a signal decay curve at a high b-value due to the low signal-to-noise ratio, which contributes to the underestimated signal decay at a high b-value.

Many mathematical models, including the monoexponential model, the intravoxel incoherent motion model (IVIM), the stretched exponential model, and diffusion kurtosis imaging, are used to fit DWI signals (15–17). However, the monoexponential model is the most commonly used model for ADC calculation in human medicine, because it is the simplest method to analyze DWI, and only two or more b-values are required to analysis (18). The ADC calculated from the monoexponential model has shown good correlation with the histopathologic degree of renal fibrosis, estimated glomerular filtration rate, and serum creatinine levels in human patients with chronic kidney diseases (3, 19). However, the ADC calculated using the monoexponential model is strongly dependent on the selected b-values, because only a small number of b-values are used in this calculation. Moreover, because the monoexponential model cannot separate the signal decay caused by capillary perfusion and tissue diffusion, the ADC reflects tissue diffusion as well as microcirculation, which may be viewed as a pseudodiffusion process mimicking diffusion (15, 16). Therefore, in kidneys with a strong microcirculatory flow, monoexponential analysis of DWI has been considered insufficient to describe the diffusion-weighted signal decay from tissue (15, 18, 19).

The signal decay caused by blood microcirculation results in a bi-exponential decay of the diffusion signal (4, 16, 17). Perfusion effects can be attenuated by acquiring data at multiple low b-values. IVIM using a bi-exponential fitting of DWI with multiple b-values allows for the calculation of a pure diffusion coefficient (D), pseudodiffusion coefficient (D^*^), and a perfusion fraction within blood vessels and tubuli (*f*_p_), and helps differentiate pure diffusion characteristics from perfusion-related pseudodiffusion. In a previous human study of renal dysfunction, it was possible to detect perfusion changes earlier by using the D^*^ value than by using changes in ADC (20). In addition, the *f*_p_ value showed a stronger correlation with histological fibrosis than did the ADC; the *f*_p_ and D had higher accuracy in differentiating enhancing mass from non-enhancing mass than ADC (8, 21).

In veterinary medicine, only two studies have evaluated ADC values in the kidney: one was performed in a canine kidney with ischemia and reperfusion injury using three b-values (0, 30, and 300 s/mm^2^), and another was performed in healthy cats with three b-values (0, 300, and 600 s/mm^2^) (22, 23). Both studies calculated ADC values using the monoexponential model, and they did not test different combinations of b values to optimize them for the ADC calculation for the kidney. Considering the effect of the selected b-values on the precision of the estimated renal DWI data, optimization of b-value sampling for DWI of the kidney is needed to minimize analytically derived error in dogs.

In this study, renal DWI was acquired with nine b-values from the bilateral renal cortex and medulla, and analyzed for the measurement of the ADC using the monoexponential model, and the IVIM parameters using the bi-exponential model. The purpose of this study was to provide reference values of the ADC, D, D^*^, and *f*_p_ of a normal kidney in healthy dogs, and to evaluate the interobserver and intraobserver reliability and test-retest repeatability of these measurements. In addition, ADC measurements with different b-value combinations consisting of three b-values each were compared to the ADC from nine b-values to evaluate the change of ADC according to b-value selection.

## Materials and Methods

The study protocol was approved by the Institutional Animal Care and Use Committee at Chonnam National University. The protocol for the care of dogs adhered to the Guidelines for Animal Experiments of Chonnam National University (CNU IACUC-YB-R-2019-68).

### Animals

Eight purpose-bred beagles, four intact male and four intact female dogs, were used in this prospective, experimental study. The median age of the dogs was 2 years (1–3 years), and the median weight was 10.2 kg (8.7–12.6 kg). All dogs were clinically healthy based on a physical examination, and an evaluation of blood pressure, complete blood count, serum biochemistry, urinalysis including urine dipstick and urine specific gravity, thoracic and abdominal radiographs, abdominal ultrasonography, and echocardiography.

### Magnetic Resonance Image Acquisition

The dogs were fasted for 24 h. Subsequently, a 20-gauge catheter was placed in the cephalic vein, and anesthesia was induced through an IV injection of 3 mg/kg of alfaxalone (Alfaxan®, 10 mg/ml, Careside, Gyeonggi-do, South Korea) in each dog. An endotracheal tube was placed, and anesthesia was maintained with isoflurane (2–4%) and oxygen (1–2 L/min). All MRI examinations were performed on dorsal recumbency using a 3.0T whole-body scanner (Achieva, Philips Healthcare, Best, Netherlands) with a 32-channel SENSE torso/cardiac coil. Three orthogonal plane images were obtained with three-dimensional T1 weighted scans as a localizer. Transverse plane DWI images were acquired using single-shot echo-planar imaging using nine different b-values (0, 50, 70, 100, 150, 200, 500, 800, and 1,000 s/mm^2^) with the following setting: TR = 10.9–14.7 s, TE = 61–67 ms, flip angle = 90°, and slice thickness = 1.8 mm. All DWI scans were performed during free-breathing while maintaining the respiratory rate at about 10 times per minute, in a regular pattern. One week later, a second set of DWI images were obtained using the same protocols. Blood analysis and urinalysis including urine dipstick and urine specific gravity tests were repeated before the second DWI scan.

### Mapping the ADC, D, D^*^, and *f*_p_ From DWI Data

**Figure 1:**
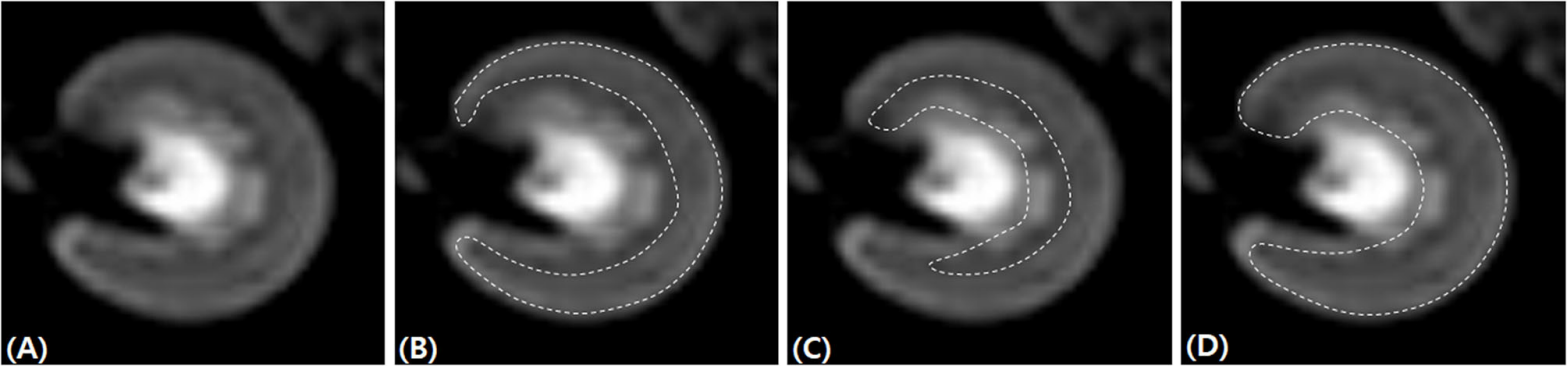
Placement of regions of interest for renal diffusion-weighted imaging analysis. The left kidney at the hilar level on transverse diffusion-weighted imaging images with b = 0 s/mm^2^ **(A)**. The regions of interest (dashed line) were traced manually over the renal cortex **(B)**, outer medulla 596 **(C)**, and the entire kidney **(D)**.

DWI data were post-processed using the diffusion analysis software (EXPRESS, Philips Healthcare, Seoul, Korea). The ROIs were traced manually over the renal cortex, outer medulla, and the entire kidney at the hilar level of the kidney, on transverse diffusion images with b = 0 s/mm^2^ (Figure 1). For each dog, ROIs were drawn in the left and right kidneys while carefully excluding the renal sinus, vascular structures, tissue boundaries, and artifacts. The signal decay curves were obtained by fitting the appropriate signal attenuation models.

The ADC value was computed by the monoexponential fitting of the mean ROI signal intensity (Equation 1):

(1) $$S\left(b\right)=S_{0}e^{-bADC}$$

where S(b) corresponds to the mean signal intensity on DWI with a certain b-value, and S(0) is the mean signal intensity on DWI with a b-value = 0 s/mm^2^.

IVIM diffusion data such as D, D^*^, and *f*_p_ were computed using the bi-exponential model (Equation 2):

(2) $$S\left(b\right)=f_{p}S_{0}e^{-bD^{*}}\;+\;\left(1-\;f_{p}\right)S_{0}e^{-bD}$$

To calculate the IVIM parameters, a two-step approach was used. First, a threshold of b-value was set at 200 s/mm^2^, and Equation 2 was simplified as Equation 3, because D^*^ can be neglected at a high b-value (> 200 s/mm^2^):

(3) $$S\left(b\right)=\;\left(1-\;f_{p}\right)S_{0}e^{-bD}$$

The D value was determined from the monoexponential fit using Equation 3. Second, the D^*^ and *f*_p_ were obtained from Equation 2 using the D value calculated in the first step. A color-coded map of the ADC, D, D^*^, and *f*_p_ was obtained with Gaussian smoothing using the software.

### ADC Values According to the b-Value Combinations

ADC measurement was repeated 10 times using 10 different combinations consisting of three b-values each: combinations consisting of low b-values (B1, B2), intermediate b-values (B3, B4), high b-values (B5, B6), and a combination of these b-value types (B7, B8, B9, B10) (Table 1). The average ADC value of both kidneys obtained from 10 different b-value combinations were compared with the ADC values obtained using nine b-values (B0).

**Table 1:** Different b-value combinations for ADC measurement.

**b-value combination**	**b-values (s/mm^**2**^)**
B0	0, 50, 70, 100, 150, 200, 500, 800, 1,000
B1	0, 50, 100
B2	0, 100, 200
B3	0, 200, 500
B4	0, 200, 800
B5	0, 500, 1,000
B6	0, 800, 1,000
B7	0, 50, 800
B8	0, 100, 800
B9	0, 50, 1,000
B10	0, 100, 1,000

### Reproducibility and Repeatability of DWI

The first set of DWI images was evaluated by a fourth-year PhD student (S.K.L.) and one veterinarian (J.R.L) with 1 year of radiology experience. Two observers who were blinded to each other's assessment measured DWI parameters individually, and interobserver reliability was assessed. After at least a 7-day interval, the evaluation of the first set of DWI images was repeated by observer 1 (S.K.L), who was blinded to the previous result, and intraobserver reliability was assessed. The second set of DWI images was analyzed by observer 1, who was blinded to the first set of DWI data, and the repeatability of DWI measurement was assessed. The first set of DWI measurements obtained by observer one were used for the comparison of ADC and IVIM parameters between the left and right kidneys and between the renal cortex and medulla, and for the evaluation of the difference in ADC values according to the b-value combinations.

### Statistical Analysis

Statistical analyses were performed using the SPSS program (IBM SPSS Statistics 25, IBM, Corporation, NY, USA). The Kolmogorov-Smirnov test was performed to determine the normality of the data. Data are presented as mean and standard deviation. Intraobserver and interobserver reliability of ADC and IVIM parameters were evaluated by calculating the intraclass correlation coefficient (ICC). The repeatability of DWI MRI measurements was evaluated by calculating the coefficient of variation (CV) and by Bland-Altman analysis including bias and 95% limits of agreements. The following criteria were used for analyzing the ICC: excellent (≥0.90), good (= 0.75 to 0.89), fair (0.50 to 0.74), and poor (<0.50) (24). The interpretation of CV was according to the following definitions: excellent (<10%), good (10 to 20%), acceptable (21 to 30%), and poor (>30%) (25). Because all data showed normal distributions, a paired *t*-test was used for analyzing the difference in the ADC, D, D^*^, and *f*_p_ between the first and second scans, between the renal cortex and medulla in each kidney, and between the left and right kidneys in each dog. The ADC values obtained from 10 combinations consisting of three b-values each were compared with the ADC values obtained from all nine b-values, using a paired *t*-test. The 95% confidence interval of the ADC, D, D^*^, and *f*_p_ was calculated for providing the reference range. The level of significance for all tests was set as p < 0.05.

## Results

In total, 16 DWI scans were performed successfully in eight beagle dogs. The mean DWI MRI scan duration was 13 min 55 s. Renal DWI showed good corticomedullary differentiation to assist the manual tracing of ROIs on the diffusion images. The renal cortex and inner stripe of the outer medulla showed hyperintense to the renal cortex, and the inner medulla had higher signal intensity on transverse diffusion images with b = 0 s/mm^2^. There was no severe image distortion from artifacts such as susceptibility and motion artifacts in any dog.

### Mapping the ADC, D, D^*^, and *f*_p_ From DWI Data

Table 2 shows the ADC, D, D^*^, and *f*_p_ values of the renal cortex, medulla, and the entire kidney in both kidneys, measured from the first set of DWI images. The ADC value was significantly higher in the renal cortex than renal medulla in both kidney, and D value was significantly higher in the left renal cortex than in left renal medulla (Table 3). The mean value of ADC, D, and *f*_p_ was significantly higher in the renal cortex than renal medulla. There was no significant difference in D^*^ between the renal cortex and medulla. There was no significant difference in any of the parameters between the left and right kidneys in each dog.

**Table 2:** The ADC, D, D^*^ and *f*_p_ values measured from the renal cortex and medulla, and the entire kidney, derived from IVIM analysis in healthy beagle dogs.

**Region**	**Parameter**	**Cortex**	**Medulla**	**Entire kidney**
Left kidney	ADC (10^−3^ mm^2^/s)	1.97 ± 0.18 (1.85–2.08)	1.83 ± 0.12 (1.75–1.91)	1.87 ± 0.12 (1.79–1.95)
	D (10^−3^ mm^2^/s)	1.53 ± 0.17 (1.42–1.64)	1.44 ± 0.12 (1.36–1.52)	1.47 ± 0.13 (1.38–1.55)
	D* (10^−3^ mm^2^/s)	17.07 ± 3.95 (14.49–19.65)	17.06 ± 4.59 (14.06–20.06)	16.84 ± 3.59 (14.49–19.18)
	*f*_p_ (%)	23.72 ± 6.24 (19.64–27.79)	23.41 ± 4.96 (20.17–26.65)	23.50 ± 5.41 (19.97–27.03)
Right kidney	ADC (10^−3^ mm^2^/s)	1.94 ± 0.17 (1.83–2.06)	1.77 ± 0.15 (1.68–1.87)	1.84 ± 0.14 (1.75–1.93)
	D (10^−3^ mm^2^/s)	1.51 ± 0.09 (1.45–1.56)	1.43 ± 0.10 (1.36–1.50)	1.45 ± 0.08 (1.39–1.50)
	D* (10^−3^ mm^2^/s)	14.32 ± 4.13 (11.62–17.02)	16.55 ± 6.54 (12.28–20.82)	14.79 ± 4.36 (11.94–17.64)
	*f*_p_ (%)	24.27 ± 3.72 (21.84–26.69)	21.28 ± 5.15 (17.92–24.65)	23.01 ± 3.99 (20.41–25.61)
Mean value	ADC (10^−3^ mm^2^/s)	1.96 ± 0.17 (1.84–2.07)	1.80 ± 0.13 (1.72–1.89)	1.85 ± 0.13 (1.77–1.94)
	D (10^−3^ mm^2^/s)	1.52 ± 0.12 (1.44–1.60)	1.44 ± 0.10 (1.37–1.50)	1.46 ± 0.09 (1.40–1.52)
	D* (10^−3^ mm^2^/s)	15.69 ± 2.61 (13.99–17.40)	16.80 ± 3.09 (14.79–18.82)	15.81 ± 2.07 (14.46–17.17)
	*f*_p_ (%)	23.99 ± 4.56 (21.01–26.97)	22.34 ± 4.75 (19.24–25.45)	23.26 ± 4.42 (20.37–26.14)

The data are presented as mean with standard deviation, and 95% confidence interval. ADC, apparent diffusion coefficient; D, pure diffusion coefficient; D^*^, pseudodiffusion coefficient; fp, perfusion fraction.

**Table 3:** Comparison of the ADC, D, D^*^, and *f*_p_ between the renal cortex and medulla, and between the left and right kidneys.

**Comparison**	**Site**	***P*****-value for each parameter**			
		**ADC**	**D**	D*	***f*_**p**_**
Cortex vs. Medulla	Left kidney	0.035	0.019	0.995	0.774
	Right kidney	0.012	0.081	0.194	0.063
	Mean value	0.018	0.026	0.289	0.047
Left kidney vs. Right kidney	Cortex	0.475	0.639	0.276	0.767
	Medulla	0.091	0.732	0.891	0.146
	Entire kidney	0.290	0.710	0.454	0.720

ADC, apparent diffusion coefficient; D, pure diffusion coefficient; D^*^, pseudodiffusion coefficient; fp, perfusion fraction.

### ADC Values According to the b-Value Combinations

**Figure 2:**
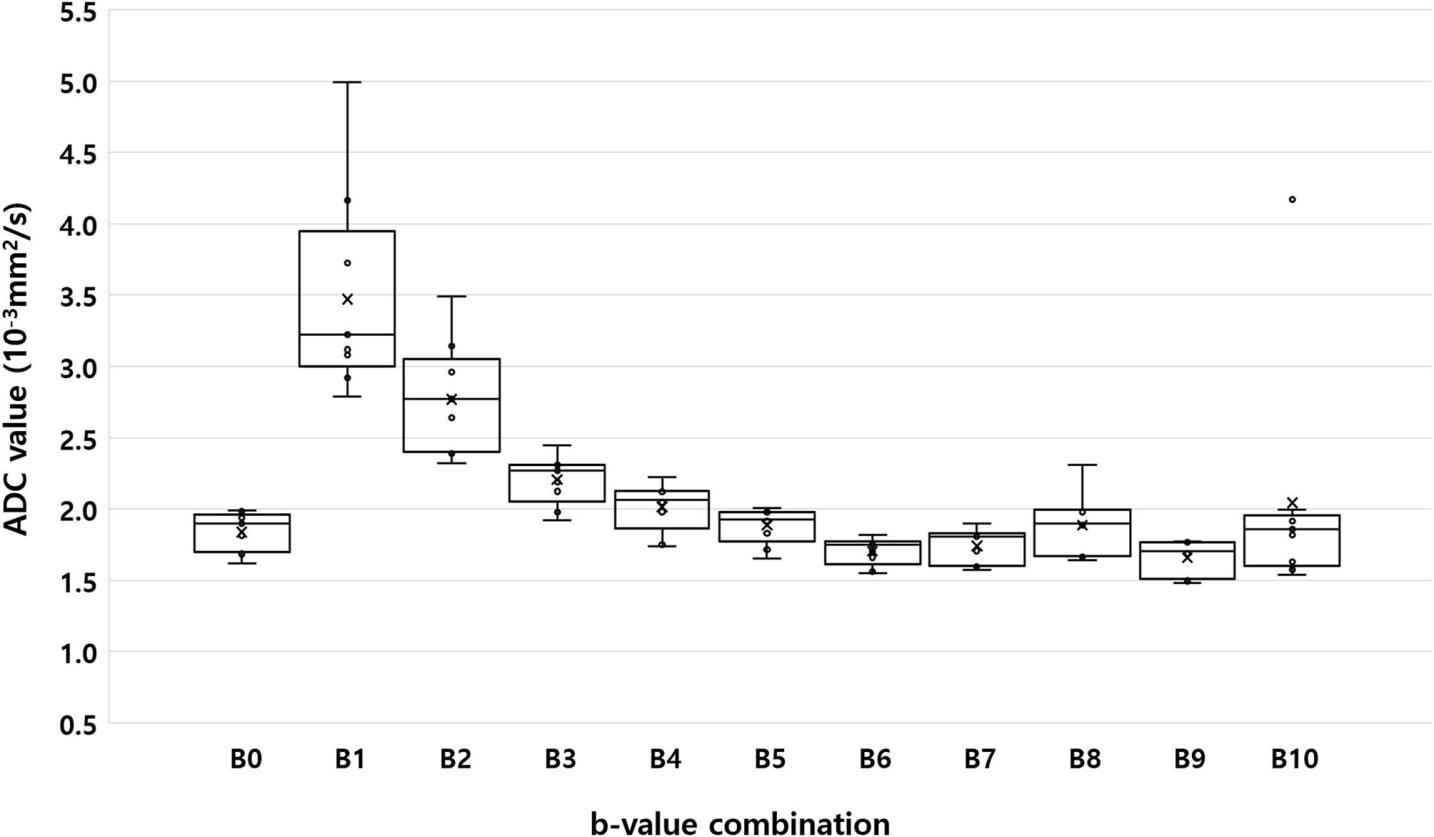
Box-plot of median apparent diffusion coefficient calculated using the monoexponential model with nine b-values (B0; b = 0, 50, 70, 100, 150, 200, 500, 800, and 1,000 s/mm^2^), and 10 different b-value combinations consisting of three b-values each (B1: b = 0, 50, and 100 s/mm^2^; B2: b = 0, 100, and 200 s/mm^2^; B3: b = 0, 200, and 500 s/mm^2^; B4: b = 0, 200, and 800 s/mm^2^; B5: b = 0, 500, and 1,000 s/mm^2^; B6: b = 0, 800, and 1,000 s/mm^2^; B7: b = 0, 50, and 800 s/mm^2^; B8: b = 0, 100, and 800 s/mm^2^; B9: b = 0, 50, and 1,000 s/mm^2^; and B10: b = 0, 100, and 1,000 s/mm^2^). Within each box, the horizontal line marks the median value. Boxes extend from the 25^th^ to the 75^th^ percentile of each value's distribution. Vertical extending lines denote the minimum and maximum values.

Figure 2 shows the ADC values of the renal parenchyma according to the combination of b-values. Compared to ADC values calculated using nine b-values (B0), the calculated ADC was significantly overestimated in B1, B2, and B3 which did not involve the use of high b-values (800 or 1,000 s/mm^2^). In contrast, when using only high b-values like in B6 (0, 800, and 1,000 s/mm^2^), or when using a combination of a very low b-value (50 s/mm^2^) with a high b-value (B7; 800 s/mm^2^ or B9; 1,000 s/mm^2^), the ADC was significantly underestimated compared to the ADC values obtained using nine b-values. Among the b-value combinations, the ADC value obtained from B8 (0, 100, and 800 s/mm^2^) had the highest ICC with the ADC obtained with B0 (Table 4).

**Table 4:** ADC values of the renal parenchyma according to the combination of b-values.

**b-value combinations**	**ADC (10^**−3**^mm^**2**^/s)**	**vs. ADC_**B0**_**	**Intraclass correlation coefficient with ADC**_****B0****_	
**B_**x**_ (s/mm^**2**^)**	**Mean ± SD**	**p-value**	**ICC (95% CI)**	***P*-value**
B0	1.84 ± 0.13	–	–	–
B1 (0, 50, 100)	3.47 ± 0.67	0.008	0.054 (−0.110–0.428)	0.323
B2 (0, 100, 200)	2.77 ± 0.36	0.000	0.140 (−0.088–0.570)	0.092
B3 (0, 200, 500)	2.21 ± 0.16	0.000	0.401 (−0.021–0.835)	0.000
B4 (0, 200, 800)	2.02 ± 0.16	0.000	0.708 (−0.133–0.944)	0.000
B5 (0, 500, 1,000)	1.89 ± 0.12	0.136	0.863 (0.432–0.968)	0.003
B6 (0, 800, 1,000)	1.70 ± 0.09	0.000	0.706 (−0.135–0.944)	0.000
B7 (0, 50, 800)	1.74 ± 0.12	0.000	0.850 (−0.145–0.975)	0.000
B8 (0, 100, 800)	1.89 ± 0.20	0.263	0.878 (0.508–0.972)	0.003
B9 (0, 50, 1,000)	1.66 ± 0.11	0.000	0.663 (−0.032–0.936)	0.000
B10 (0, 100, 1,000)	2.04 ± 0.77	0.514	0.316 (−2.207–0.848)	0.015

*B0 is a combination of nine b-values (0, 50, 70, 100, 150, 200, 500, 800, and 1,000 s/mm^2^). The data are presented as the mean and standard deviation of ADC values. ADC, apparent diffusion coefficient; CI, confidence interval; ICC, intraclass correlation coefficient; SD, standard deviation*.

### Reliability and Repeatability of the ADC and IVIM Parameters

Table 5 shows the ICC for intraobserver and interobserver reliability of the ADC, D, D^*^, and *f*_p_. Most of the parameters showed an excellent degree of interobserver and intraobserver reliability, while some parameters showed good reliability. Table 6 summarizes the difference, CVs, and Bland-Altman agreement among the ADC, D, D^*^, and *f*_p_ values in the bilateral renal cortex and medulla between the first and second scans. There was no significant test-retest difference in the ADC, D, D^*^, and *f*_p_. The ADC and D had excellent repeatability, and the D^*^ and *f*_p_ had good to excellent repeatability.

**Table 5:** Intraclass correlation coefficient values for intraobserver and interobserver reliability of the ADC, D, D^*^, and *f*_p_.

**Analysis**	**Measurement site**		**Intraclass correlation coefficient (95% CI) for each parameter**			
			**ADC**	**D**	D*	***f*_**p**_**
**Interobserver**	Left kidney	Cortex	0.909 (0.547–0.982)	0.973 (0.863–0.995)	0.944 (0.722–0.989)	0.955 (0.773–0.991)
		Medulla	0.858 (0.289–0.971)	0.969 (0.843–0.994)	0.886 (0.429–0.977)	0.941 (0.703–0.988)
		Entire kidney	0.926 (0.630–0.985)	0.979 (0.896–0.996)	0.937 (0.685–0.987)	0.970 (0.849–0.994)
	Right kidney	Cortex	0.981 (0.905–0.996)	0.987 (0.935–0.997)	0.948 (0.738–0.990)	0.942 (0.713–0.988)
		Medulla	0.984 (0.918–0.997)	0.948 (0.741–0.990)	0.963 (0.815–0.993)	0.959 (0.794–0.992)
		Entire kidney	0.993 (0.967–0.999)	0.956 (0.782–0.991)	0.987 (0.935–0.997)	0.951 (0.754–0.990)
**Intraobserver**	Left kidney	Cortex	0.942 (0.709–0.988)	0.984 (0.922–0.997)	0.777 (−0.112–0.955)	0.988 (0.940–0.998)
		Medulla	0.982 (0.910–0.996)	0.969 (0.846–0.994)	0.873 (0.367–0.975)	0.889 (0.445–0.978)
		Entire kidney	0.974 (0.869–0.995)	0.996 (0.981–0.999)	0.851 (0.255–0.970)	0.992 (0.959–0.998)
	Right kidney	Cortex	0.965 (0.825–0.993)	0.755 (−0.222–0.951)	0.957 (0.787–0.991)	0.932 (0.659–0.986)
		Medulla	0.981 (0.906–0.996)	0.874 (0.371–0.975)	0.914 (0.569–0.983)	0.934 (0.672–0.987)
		Entire kidney	0.975 (0.876–0.995)	0.866 (0.332–0.973)	0.944 (0.720–0.989)	0.944 (0.722–0.989)

ADC, apparent diffusion coefficient; CI, confidence interval; D, pure diffusion coefficient; D^*^, pseudodiffusion coefficient; fp, perfusion fraction.

**Table 6:** Difference, coefficient of variance, and Band-Altman agreement of the ADC, D, D^*^, and *f*_p_ in bilateral renal cortex and medulla between the first and second scans in healthy dogs.

**Measurement site**		**Parameters**	**Mean** **±** **SD**		***P*-value**	**CV (%)**	**Bland-Altman analysis**
			**1^**st**^ scan**	**2^**nd**^ scan**			**Bias (95% LOA)**
Left kidney	Cortex	ADC (10^−3^ mm^2^/s)	1.97 ± 0.18	1.93 ± 0.20	0.755	5.05	−0.03 (−0.51–0.45)
		D (10^−3^ mm^2^/s)	1.53 ± 0.17	1.50 ± 0.07	0.554	4.20	−0.04 (−0.35–0.27)
		D* (10^−3^ mm^2^/s)	17.07 ± 3.95	15.64 ± 4.01	0.285	10.12	−1.43 (−7.83–4.97)
		*f*_p_ (%)	23.72 ± 6.24	23.65 ± 4.37	0.983	12.75	−0.06 (−15.45–15.33)
	Medulla	ADC (10^−3^ mm^2^/s)	1.83 ± 0.12	1.91 ± 0.23	0.509	5.73	0.07 (−0.48–0.63)
		D (10^−3^ mm^2^/s)	1.40 ± 0.12	1.48 ± 0.09	0.571	4.76	0.03 (−0.25–0.32)
		D* (10^−3^ mm^2^/s)	17.06 ± 4.59	15.66 ± 5.14	0.504	15.57	−1.39 (−11.66–8.88)
		*f*_p_ (%)	23.41 ± 4.96	23.95 ± 6.01	0.867	16.00	0.54 (−15.44–16.51)
	Entire kidney	ADC (10^−3^ mm^2^/s)	1.87 ± 0.12	1.89 ± 0.18	0.803	4.34	0.02 (−0.38–0.42)
		D (10^−3^ mm^2^/s)	1.47 ± 0.13	1.48 ± 0.08	0.797	4.30	0.01 (−0.25–0.28)
		D* (10^−3^ mm^2^/s)	16.84 ± 3.59	15.44 ± 4.03	0.255	9.58	−1.40 (−7.25–4.45)
		*f*_p_ (%)	23.50 ± 5.41	23.66 ± 4.38	0.954	13.25	0.16 (−13.49–13.81)
Right kidney	Cortex	ADC (10^−3^ mm^2^/s)	1.94 ± 0.17	1.98 ± 0.28	0.604	3.47	−0.21 (−1.60–1.17)
		D (10^−3^ mm^2^/s)	1.51 ± 0.09	1.43 ± 0.11	0.195	3.52	−0.07 (−0.34–0.19)
		D* (10^−3^ mm^2^/s)	14.32 ± 4.13	13.57 ± 3.08	0.675	10.91	−0.75 (−9.64–8.14)
		*f*_p_ (%)	24.27 ± 3.72	26.95 ± 7.39	0.322	12.39	2.69 (−10.39–15.76)
	Medulla	ADC (10^−3^ mm^2^/s)	1.77 ± 0.15	1.78 ± 0.12	0.836	3.68	−0.22 (−1.47–1.03)
		D (10^−3^ mm^2^/s)	1.43 ± 0.10	1.36 ± 0.15	0.250	4.74	−0.07 (−0.35–0.21)
		D* (10^−3^ mm^2^/s)	16.55 ± 6.54	13.47 ± 3.68	0.337	19.21	−3.08 (−18.59–12.43)
		*f*_p_ (%)	21.28 ± 5.15	25.04 ± 4.81	0.224	16.13	3.76 (−10.85–18.36)
	Entire kidney	ADC (10^−3^ mm^2^/s)	1.84 ± 0.14	1.82 ± 0.14	0.751	3.15	−0.02 (−0.29–0.25)
		D (10^−3^ mm^2^/s)	1.45 ± 0.08	1.37 ± 0.12	0.207	4.66	−0.08 (−0.37–0.22)
		D* (10^−3^ mm^2^/s)	14.79 ± 4.36	12.25 ± 1.09	0.152	12.92	−2.54 (−10.73–5.65)
		*f*_p_ (%)	23.01 ± 3.99	25.96 ± 3.40	0.139	9.65	2.95 (−6.20–12.10)

ADC, apparent diffusion coefficient; CV, coefficient of variation; D, pure diffusion coefficient; D*, pseudodiffusion coefficient; f_p_, perfusion fraction; LOA, limit of agreement; SD, standard deviation.

## Discussion

In this study, renal DWI was acquired with nine b-values; the ADC was analyzed using the monoexponential model, and the IVIM parameters were analyzed using the bi-exponential model. This is the first study to apply IVIM-DWI with multiple b-values to the kidney, and provides both ADC and IVIM parameters of the bilateral renal cortex, medulla, and the entire kidney in healthy dogs. Both the ADC and IVIM parameters were reproducible in healthy dogs, although the repeatability of the perfusion-related parameters (D^*^ and *f*_p_) was slightly lower. Among the different b-value combinations, the ADC obtained with b = 0, 100, and 800 s/mm^2^ had the best agreement with the ADC obtained with nine b-values.

The values of the ADC and the IVIM parameters measured in this study were similar to those measured in humans with a similar b-value distribution (number of b-values = 11; distribution = 0 to 1,000 s/mm^2^), although there is no renal DWI study in healthy dogs (26). Diffusion analysis can be performed by the placement of ROIs in the renal cortex and medulla. Additionally, the placement of ROIs including the entire kidney or the corticomedullary junction is essentially used when the corticomedullary junction is indistinct due to low spatial resolution, or in chronic renal diseases (27, 28). Regardless of ROI placement, DWI was able to estimate renal fibrosis and diagnose kidney disease or renal dysfunction with high sensitivity in previous studies (6, 9, 10, 27, 29). This study provides the ADC and the IVIM parameters of the renal cortex and medulla, and from the entire kidney; these parameters can be used as reference data in dogs with diffuse kidney diseases such as chronic kidney disease, where the renal corticomedullary border is lost.

In the kidney, compared to ADC analysis, IVIM analysis is considered to be more informative for assessing renal function and microstructure, because it can differentiate between signals due to molecular diffusion and those due to capillary perfusion (8, 9, 20, 21, 30). The D value is a similar to the ADC, but unlike the ADC, it reflects pure tissue diffusion while excluding perfusion. The D value has a strong correlation with the ADC, but is lower than the ADC. In patients with kidney diseases, the D value is generally decreased according to the decrease in diffusion caused by microstructural changes such as cellular swelling (8, 31). The *f*_p_ and D^*^ values are perfusion-related parameters, but do not simply reflect the microcirculation of blood. Tubular flow and water reabsorption also contribute to microscopic flow in renal tissue, and influence the *f*_p_ and D^*^ (32, 33). The *f*_p_ refers to the proportion of vascular and tubular fluid volume to the total fluid in the tissue, which is related to total flow (33). In previous studies, the *f*_p_ was reported to correlate with renal blood flow obtained from arterial spin labeling and dynamic contrast-enhanced imaging (31, 34). The D^*^ value reflects the average velocity of vascular blood and tubular fluid within the kidney (33). However, the main determining factor of the D^*^ is controversial. In some studies, the D^*^ was sensitive to vascular blood, but other studies failed to find a correlation between the D^*^ and renal blood flow; one author suggested that the D^*^ was mainly determined by tubular fluid rather than by microcirculation perfusion (11, 31, 34).

The differences in IVIM parameters between the renal cortex and medulla may be related to the renal microstructure and microcirculation. The renal cortex had a higher blood volume, a larger tubular diameter, and a smaller proportion of interstitium than the renal medulla (35, 36). In most human studies, the ADC, D, and *f*_p_ are higher in the renal cortex than in the renal medulla (25, 26, 33, 37, 38). Similarly, in this study, the ADC, D, and *f*_p_ tended to be higher in the cortex than in the medulla, though the difference was not significant. The higher ADC values of the cortex may reflect higher perfusion in the cortex. The higher D value in the cortex implies a higher diffusion in the cortex, and it is thought that the difference in diffusion between the cortex and medulla may be caused by the difference in the microstructure, such as that in the tubules, interstitium, and vessels. Larger blood and tubular fluid volume in the cortex may contribute to higher *f*_p_ of the cortex. The D^*^ would be higher in the cortex than in the medulla because this parameter reflects perfusion. However, the D^*^ in this study tended to be higher in the medulla than in the cortex, although the difference was not significant. There is no clear trend for differences in the D^*^ according to the renal cortex and medulla in humans, but the D^*^ was higher in the medulla than in the cortex in some studies (14, 26, 38). This result cannot be clearly explained, but one author suggested that it may be attributed to the fast tubular flow velocity in the loop of Henle (33).

In this study, the ADC and the IVIM parameters were not different between the two kidneys, as reported in humans and animal models (9, 10, 39). Several previous studies used the average values of both kidneys for statistical analysis on the assumption that there is no difference in the values between the two kidneys (6, 9, 28, 37). Similarly, we used the average value of the entire both kidney for comparing ADC values according to the b-value combinations to minimize the effect of ROI.

Selecting the optimal b-value for renal DWI is important, because the ADC varies depending on the b-value selected (37). Although the ideal number and choice of b-values for renal DWI has not been established yet even in humans, a consensus for renal DWI recommends the use of a larger number of b-values for accurate ADC measurement rather than the use of two b-values (18). Therefore, this study used a combination of three b-values to determine a valid combination for the accurate estimation of ADC in renal DWI. Among the different b-value combinations, the ADC from a b-value combination of 0, 100, and 800 s/mm^2^ had the highest ICC with the ADC obtained using nine b-values. This b-value combination was consistent with the recommendation for renal DWI in humans that uses a b-value of <200 s/mm^2^ as the low b-value, and 800 s/mm^2^ or 1,000 s/mm^2^ as the maximum b-value for ADC measurement (18, 40). The ADC was underestimated when using high b-values alone in this study, which may be related with the prominent non-Gaussian diffusion and decreased signal-to-noise at high b-values compared to that at lower b-values (16). Meanwhile, the ADC was markedly overestimated when only low b-values were used, because perfusion has a significant contribution to signal loss at low b-values (15, 37). The signal decay by perfusion may be weak at very low b-values when a very low b-value (50 s/mm^2^) was used with a high b-value (800 or 1,000 s/mm^2^), which contributes to the underestimation of the ADC compared to the ADC obtained from the nine b-values in this study. In a previous study in dogs with renal injury associated with ischemia/reperfusion, the ADC was measured using three b-values (0, 30, and 300 s/mm^2^) before ischemia induction (23). The ADC value was higher than ours, which may be due to overestimation related to the use of a low b-value combination.

The optimal number and distribution of b-values for IVIM analysis for the kidney have not been reported in literature. Perfusion analysis using the IVIM model needs more b-values and appropriate selection of the b-value distribution to sample the fast pseudodiffusion (b <200) and slower true diffusion (200 < b <1,000) decays (15, 18). Recent reports of human studies have recommend the use of at least six b-values, and eight or more b-values are considered to be more ideal in renal IVIM-DWI (18). When distributing b-values, it is recommended to use more low b-values than high b-values, because accurate measurement of the degree of signal attenuation over a small range of low b-values is more susceptible to errors than that of high b-values, and accurate delivery of low b-values can be challenging (15, 41). Various distributions of b-values were used previously, and 800 and 1,000 s/mm^2^ were most frequently used as the maximum b-value (8, 9, 18, 20, 27, 30, 32, 40). In this study, nine b-values were used for IVIM analysis, and five low b-values (0, 70, 100, 150, and 200 s/mm^2^) and three high b-values (500, 800, and 1,000 s/mm^2^) were selected.

In this study, almost all the ADC and D, D^*^, and *f*_p_ values had excellent interobserver and intraobserver reproducibility. This result may be attributed to the selection of consistent slices at the renal hila level for DWI analysis, and a large ROI tracing the entire kidney or cortex/medulla (in contrast to a small circular ROI). Test-retest repeatability was also excellent in the ADC and D values, while it was good to excellent in the D^*^ and *f*_p_ values. The signal measurements at low b-values are more prone to measurements errors and are more sensitive to signal-to-noise variation, and this may be associated with the lower repeatability of the D^*^ and *f*_p_ (15, 40). In addition, the high perfusion and large tubular diameter of the kidney and the experimental uncertainty related to physiological changes such as blood volume and velocity may also have contributed to the lower repeatability of D^*^ and *f*_p_ (25, 33).

The clinical utility of DWI is under investigation in patients with various kidney diseases such as CKD, renal artery stenosis, diabetic nephropathy, contrast media-induced nephropathy, and kidney transplant to evaluate renal function and predict histopathological changes. Most promising results in fibrosis estimation in CKD and the correlation between ADC and histopathological fibrosis score has been confirmed in humans and animal experiments (5, 6, 9). Compared to ADC which reflects perfusion change as well as renal fibrosis, D in IVIM DWI had a higher correlation with fibrosis and cell density than ADC in rabbits with unilateral urinary tract obstruction (29). D^*^ and *f*_p_ also showed a correlation with histopathologic fibrosis score, although they may be associated with decreased perfusion accompanied by fibrosis rather than reflecting fibrosis itself (8, 9). Because the ADC and IVIM parameters were correlated with renal functional markers such as glomerular filtration rate, creatinine, and proteinuria, DWI MRI also provided functional information in patients with CKD (6, 9).

Another potential use of DWI is the characterization of the renal mass (30, 42, 43). The usefulness of ADC has been described in differentiating benign from malignant tumor and grading and pathologic subtyping of renal carcinoma. Malignant tumors have lower ADCs than benign tumors and high-grade tumors have lower ADCs than low-grade tumors (42–44). The sensitivity and specificity of differentiating benign and malignant tumors are generally higher than 70% (42). Although there were some discrepancies, clear cell type renal carcinoma tended to have lower ADCs than other types of renal cell carcinoma, and transitional cell carcinoma tended to have lower ADCs than renal cell carcinoma (43, 44). Recent studies suggest IVIM analysis is more informative to assessing renal tumors and vigorous researches are performed for this clinical potential (30).

In humans, various anti-fibrotic drugs to prevent CKD progressions such as TGF-β antagonists, antioxidants, and inflammatory response targeting drugs are in clinical trials (45). Angiotensin-converting enzyme inhibitors and angiotensin receptor antagonists are also known to have anti-fibrotic effect in humans (46). These drugs were routinely used in dogs and cats with CKD, but their anti-fibrotic effect was not established. Because DWI MRI can be performed repeatedly, it can be used to monitor changes in renal fibrosis within a subject and may contribute to assess the anti-fibrotic effect of the drugs and develop a new therapy for managing CKD in veterinary medicine.

One of the aspects differentiating DWI MRI application in dogs from that in humans may be the anesthesia requirements. Although the effects of general anesthesia on renal physiology have not been fully understood, general anesthesia can decrease renal blood flow and function through its cardiovascular effect. Because ADC, D^*^, and *f*_p_ are perfusion-related parameters, they may be affected by changes in the renal blood flow caused by general anesthesia. Although D reflects pure diffusion, it is thought that it may be affected by tubular and vascular microstructure changes after anesthesia due to alteration of renal blood flow and function. However, the change of DWI parameters of the kidneys according to the anesthesia was not revealed. Further studies are needed to investigate the effects of type of anesthetic agent, heart rate, or blood pressure on the DWI parameters of the kidney in dogs. Previous studies showed that renal function and renal blood flow were well-maintained in healthy anesthetized dogs, so anesthesia may not have a significant impact on DWI parameters in healthy dogs (47, 48). However, dogs with kidney disease are more susceptible to anesthesia and may have significant effects. The use of anesthetic drugs no affecting the cardiovascular system would be better to minimize the effect of anesthetics on renal blood flow and DWI parameters. In this study, alfaxalone was used for anesthesia, which has minimal effect on the cardiovascular system.

There are several limitations and considerations pertaining to this study. First, there is no gold standard technique to assess tissue diffusion, thus the DWI-measured parameters cannot be verified. Therefore, it is important to use standard protocols when performing DWI. This study used the most recently recommended protocol in humans based on data accumulated in previous studies and optimization of b-value for ADC measurements was performed by comparing ADC from multiple b-values as a reference. Histopathologic evaluation of the kidney was not performed in this study because this study was performed to identify the technical feasibility of the standard DWI protocol in healthy dogs. Further studies are needed to confirm if this protocol can reflect renal fibrosis in canine kidney diseases. Second, DWI was performed in dogs with free-breathing. Motion artifacts over successive averages of the same images results in image blurring, and motion between different diffusion-weighting causes misregistration and measurement errors (49). The effects of respiratory motion can be diminished by performing a breath-hold or with the use of respiratory triggering. However, breath-hold imaging is of limited use in IVIM measurements, as IVIM requires the acquisition of images at multiple b-values. So, the total acquisition time is over several minutes, and multiple breath-hold cycles are necessary. Respiratory triggering also prolongs the acquisition times and irregular respiratory patterns can decrease image quality (9, 32). In this study, diffusion-weighted images were obtained during free-breathing to prevent an increase in the acquisition time on the basis of a previous study which reported that the ADC and the IVIM parameters of the kidney from DWI using free-breathing were not different from those obtained from DWI using respiratory triggering (50). We were able to obtain images with excellent spatial resolution which were sufficient to analyze the DWI parameters of the kidney by reducing motion artifacts; DWI scans were only performed while maintaining the respiratory rate about 10 times per minute with a regular pattern. Third, in this study, DWI parameters were only analyzed at the hilar level of the kidney on transverse plane images. Although IVIM parameters measured at the hilar level in CKD patients in previous human studies had a correlation with histological renal fibrosis score, measurements at only one slice may not represent renal pathological changes in patients with heterogeneous renal parenchymal change (9). Further studies on ROI settings for analyzing DWI parameters in dogs with kidney disease are needed. Image orientation can affect DWI analysis. In humans, either the axial or the coronal plane is typically used for renal DWI, and measurements of DWI parameters in both image planes showed fair to excellent agreement in a human study (49). The coronal plane allows for coverage of the full kidney with fewer slices. However, it is more prone to respiratory motion artifacts and has a lower signal-to-noise ratio compared to the axial plane. In this study, transverse images were obtained to minimize motion artifacts, and to increase the signal-to-noise ratio (4, 51). Fourth, this study only used a small number of subjects of similar size and age. The spatial resolution in smaller dogs or cats may need to be adapted for clinical patients. In this study, only young healthy dogs of similar age were used. Although the effect of age on ADC values is controversial, an inverse correlation is reported to exist between them (52). Considering that kidney diseases primarily affect older dogs, reference data and the usefulness of DWI in diagnosing kidney diseases should be assessed further in older dogs.

## Conclusions

Both monoexponential and IVIM analysis of DWI MRI using multiple b-values can be performed with high reproducibility and repeatability in dogs with 3.0T MRI. The results of this study provide reference data for the ADC, D, D^*^, and *f*_p_ in healthy beagle dogs. When multiple b-values are not available, a combination of b = 0, 100, and 800 s/mm^2^ may be an alternative approach for ADC measurements of the kidney in dogs

## Data Availability Statement

The raw data supporting the conclusions of this article will be made available by the authors, without undue reservation.

## Ethics Statement

The study protocol was approved by the Institutional Animal Care and Use Committee at Chonnam National University. The protocol for the care of dogs adhered to the Guidelines for Animal Experiments of Chonnam National University (CNU IACUC-YB-R-2019-68).

## Author Contributions

S-KL and JC established the experimental design. SJ and EL performed medical examination of dogs before and after DWI MRI and performed anesthesia of dogs during DWI MRI. S-KL and C-YJ performed DWI MRI. S-KL and JL analyzed the DWI data. S-KL performed statistical analysis. The manuscript was written by S-KL and was revised by JC. All authors contributed to the article and approved the submitted version.

## Conflict of Interest

The authors declare that the research was conducted in the absence of any commercial or financial relationships that could be construed as a potential conflict of interest.
